# Detection of Parent-of-Origin Effects for the Variants Associated With Behavioral Disinhibition in the MCTFR Data

**DOI:** 10.3389/fgene.2022.831685

**Published:** 2022-04-26

**Authors:** Yi-Fan Kong, Meng-Kai Li, Yu-Xin Yuan, Zi-Ying Yang, Wen-Yi Yu, Pei-Zhen Zhao, Ji-Yuan Zhou

**Affiliations:** ^1^ Department of Biostatistics, State Key Laboratory of Organ Failure Research, Ministry of Education, and Guangdong Provincial Key Laboratory of Tropical Disease Research, School of Public Health, Southern Medical University, Guangzhou, China; ^2^ Guangdong-Hong Hong-Macao Joint Laboratory for Contaminants Exposure and Health, Guangzhou, China

**Keywords:** behavioral disinhibition, genome-wide association study, parent-of-origin effects, Minnesota Center for Twin and Family Research data, rank-based inverse normal transformation

## Abstract

Behavioral disinhibition is one of the important characteristics of many mental diseases. It has been reported in literature that serious behavioral disinhibition will affect people’s health and greatly reduce people’s quality of life. Meanwhile, behavioral disinhibition can easily lead to illegal drug abuse and violent crimes, etc., which will bring great harm to the society. At present, large-scale genome-wide association analysis has identified many loci associated with behavioral disinhibition. However, these studies have not incorporated the parent-of-origin effects (POE) into analysis, which may ignore or underestimate the genetic effects of loci on behavioral disinhibition. Therefore, in this article, we analyzed the five phenotypes related to behavioral disinhibition in the Minnesota Center for Twin and Family Research data (nicotine, alcohol consumption, alcohol dependence, illicit drugs, and non-substance use related behavioral disinhibition), to further explore the POE of variants on behavioral disinhibition. We applied a linear mixed model to test for the POE at a genome-wide scale on five transformed phenotypes, and found nine SNPs with statistically significant POE at the significance level of 5 × 10^−8^. Among them, SNPs rs4141854, rs9394515, and rs4711553 have been reported to be associated with two neurological disorders (restless legs syndrome and Tourette’s syndrome) which are related to behavioral disinhibition; SNPs rs12960235 and rs715351 have been found to be associated with head and neck squamous cell carcinoma, skin cancer and type I diabetes, while both SNPs have not been identified to be related to behavioral disinhibition in literature; SNPs rs704833, rs6837925, rs1863548, and rs11067062 are novel loci identified in this article, and their function annotations have not been reported in literature. Follow-up study in molecular genetics is needed to verify whether they are surely related to behavioral disinhibition.

## Introduction

Behavioral disinhibition refers to the problematic and uncontrolled performance of impulsive behavior, and it has been observed to be an important characteristic of several neurodevelopmental and psychiatric diseases (Sharma et al., 2014). Its core feature is the inability to regulate immediate response inclinations at the expense of long-term gains or losses. There have been substantial studies consistently showing that the individuals with a high degree of behavioral disinhibition are at elevated risk for developing a broad array of behavioral disorders including substance use disorders (McGue et al., 2013). Meanwhile, behavioral disinhibition is a behavioral trait which is hypothesized to represent a general vulnerability in the development of substance use disorders, and the ability to manage immediate impulsive responses is reduced (Vrieze et al., 2013). Studies have shown that those with greater levels of disinhibition are thought to act more impulsively and to be more inclined to seek excitement, without considering the long-term consequences of their behaviors. People with a high degree of behavioral disinhibition are also more likely to use substances and have a more difficult time quitting (Iacono et al., 2008). Severe behavioral disinhibition is more generally related to increased negative health outcomes and increased mortality during the lifespan (Amirian et al., 2010; Kubzansky et al., 2011).

With the completion of the Human Genome Project, large-scale genetic data had been generated, and the research on behavioral disinhibition has also gone further to the genetic level. Some indicators were first used to quantitatively describe the degree of behavioral disinhibition, and then the relationship between single nucleotide polymorphisms (SNPs) and these quantitative indicators related to behavioral disinhibition were explored, aiming to capture the heritable variations which affect some behaviors including dangerous behaviors and impulsive behaviors, and finally they were utilized for discovering genetic loci which are likely to cause behavioral disinhibition disorders. The twin studies found that most of the genetic influences on individual-level behavioral disinhibition disorders can be attributed to genetic influences at the level of general factors, and the heritability of the behavioral disinhibition is estimated to be between 60% and 80% (Young et al., 2000; Krueger et al., 2002; Kendler et al., 2003). Some studies have pointed out that certain genes have common effects on multiple indicators of behavioral inhibition. For example, genetic variations affecting the γ-aminobutyric acid are related to the general addiction process (Krystal et al., 2006). Agrawal et al. (2006) have shown that the SNPs in the *GABRA2* receptor gene are associated with severe addiction. Corley et al. (2008) also confirmed that the *GABRA2* receptor gene is significantly associated with a variety of drug abuse and antisocial behaviors. Similarly, the mutations in the cholinergic muscarinic receptor 2 gene are associated with the risk for smoking (Mobascher et al., 2010). Vrieze et al. (2013) used the genome-wide complex trait analysis to estimate the aggregate genetic effect between 515,384 SNPs and behavioral disinhibition, and the estimated aggregated SNPs can explain 10%∼30% of the variances in the corresponding traits. McGue et al. (2013) analyzed five quantitative indicators related to behavioral disinhibition, i.e., “nicotine,” “alcohol consumption,” “alcohol dependence,” “illicit drugs,” and “non-substance use related behavioral disinhibition” (BD), and found that SNP rs1868152 is statistically significantly related to illegal drug abuse. At the same time, by adjusting the threshold of the *p*-values of genome-wide association study (GWAS), 13 candidate SNPs which may be related to behavioral disinhibition were found. Derringer et al. (2015) used 1,901 adolescents for the GWAS of behavioral disinhibition. No single SNP was found to be significantly associated with behavioral disinhibition. However, in the subgroup analysis, it was estimated that 49.3% of the variance in behavioral disinhibition within the Caucasian sub-sample could be explained by the genetic variations, and seven genes were identified to be significantly associated with behavioral disinhibition.

However, GWAS usually regards the alleles inherited from the mother and the father as equivalent, and generally does not consider parent-of-origin effects (POE). POE is an important epigenetic phenomenon, which means that the parental source of the chromosome where the gene is located determines whether the gene is expressed. One of the important mechanisms of parental effects is genomic imprinting. Genes with imprinting effects have parental specificity in their expression. For example, paternal imprinting means that the gene inherited from the father is not expressed and only the gene inherited from the mother is expressed, which is also known as maternally derived effect. On the contrary, maternal imprinting indicates that the gene has a paternally derived effect. Different imprinting effects may lead to different diseases. For example, Prader-Willi syndrome and Angelman syndrome are both caused by the loss of the functional alleles of the genes within the imprinted region of Chromosome 15q11-13. Among them, inheriting a loss of function mutation for the *SNRPN* gene from the father can cause Prader-Willi syndrome, while inheriting a loss of function mutation for the *UBE3A* gene from the mother results in Angelman syndrome (Falls et al., 1999; Peters, 2014). Morison et al. (2001) built a database of imprinted genes (http://igc.otago.ac.nz/), and so far there are 355 records related to humans. Similarly, in the geneimprint and Otago imprint databases (https://www.geneimprint.com/), more than 150 imprinted genes have been described in humans, but there may be more imprinted genes which have not been verified (Benonisdottir et al., 2016). Many imprinted genes are essential to the normal growth, neurodevelopment, metabolism of the fetus and adult behavior (Smith et al., 2006). Imprinted mutants can affect the growth and development of individuals and cause various diseases, such as Prader-Willi syndrome, Angelman syndrome, Turner’s syndrome (Skuse et al., 1997), etc.

Although GWAS studies have demonstrated that genetic variations could explain part of the variances of the traits related to behavioral disinhibition, a GWAS of adolescent behavioral disinhibition (Derringer et al., 2015) used the restricted maximum likelihood method to estimate the proportions of the variances that can be explained by SNPs in the phenotypes, and the results showed that there is still part of genetically related variance in the residuals of the model and current GWAS results may underestimate the effect of SNPs on behavioral disinhibition. Other more complicated genetic models need to be considered to explore the realistic effect of SNPs on behavioral disinhibition. The earliest and strongest evidence for the POE on behavioral disinhibition comes from studies of alcohol dependence. Almasy and Borecki (1999) summarized the analyses in Genetic Analysis Workshop 11 based on the data from the Collaborative Study on the Genetics of Alcoholism. They discovered that more significant loci could be found in the samples split by parental origin than in the total sample, and more effects could be seen for paternal transmission than for maternal transmission. Likewise, the findings of Macciardi et al. (1999) showed that the mode of inheritance for alcoholism is probably more complex than traditional Mendelian disorders, and there are significant differences in alcoholism by gender, parent-of-origin effects and other epidemiological factors. Strauch et al. (2005) used 93 Caucasian pedigrees of the Collaborative Study on the Genetics of Alcoholism dataset and found that some loci on Chromosomes 1, 2, 10, 12, 13, 15 and 21 have paternal imprinting on alcohol dependence, and a tendency to maternal imprinting was observed at two loci on Chromosome 7. Moreover, a recent GWAS of the addiction explained that some genes, which were identified as risk factors of smoking, show an unbalanced expression of alleles biased towards paternal alleles, which may be caused by paternally derived effect (Kozlova et al., 2021). On the other hand, more and more studies have claimed that some genes with imprinting effects have a significant impact on psychiatric disorders, such as schizophrenia and bipolar disorder. For example, Ludwig et al. (2009) determined the role of the gene *LRRTM1* in the development of schizophrenia/schizoaffective disorder. In their study of 180 parent-offspring trios with schizophrenia, they found that *LRRTM1* has maternal imprinting in schizophrenia. Similarly, some genes that have been shown to be imprinted, such as *ZDBF2*, *PPP1R9A* and *DLGAP2* (Nakabayashi et al., 2004; Luedi et al., 2007), were also found to be genetic causes of schizophrenia or bipolar disorder (Li et al., 2014; Konopaske et al., 2015). Behavioral disinhibition, as an important feature of several neurodevelopmental and psychiatric disorders, may also be influenced by certain imprinted genes. However, there has been currently no POE on behavioral disinhibition systematically discussed in literature.

Therefore, in this article, we explored the POE for behavioral disinhibition based on the data of the Minnesota Center for Twin and Family Research (MCTFR) Genome-Wide Association Study of Behavioral Disinhibition. We applied a linear mixed model to examine the associations of maternally and paternally derived minor alleles with five quantitative indicators of behavioral disinhibition at a genome-wide scale so that the parental sources of genetic variants can be included in the analysis to further reveal the genetic factors which may be missed or underestimated in traditional GWAS. The purpose of this article is to propose new insights into the underlying genetic etiology of behavioral disinhibition, and to identify some novel candidate SNPs for future related researches on behavioral disinhibition in molecular genetics.

## Materials and Methods

### Samples

We used the dataset from the MCTFR Genome-Wide Association Study of Behavioral Disinhibition, which is made available from the database of Genotypes and Phenotypes (dbGaP) with accession number 86747-6 (https://www.ncbi.nlm.nih.gov/projects/gap/cgi-bin/study.cgi?study_id=phs000620.v1.p1). It is a large, ongoing and family-based epidemiological study on substance abuse and related psychopathology, consisting of three cohorts: 1) 17-year-old twins born in Minnesota from 1972 to 1979 and their parents, 2) 11-year-old twins born in Minnesota from 1977 to 1984 and their parents (later, more 11-year-old twins born from 1988 to 1994 and their parents were added), and 3) 15-year-old full biological siblings, adopted siblings, and mixed siblings (one being biologically related to the parents and the other being an adopted child) born in Minnesota from 1978 to 1988 and their parents (Iacono et al., 2006). McGue et al. (2013) conducted a genome-wide association study of behavioral disinhibition using 2,300 Caucasian families and 7,188 individuals for all the three cohorts in MCTFR. As one of the principal investigators of MCTFR, McGue M. uploaded the data of 2,183 families and 6,784 individuals to the dbGaP database for public research. Since the detection of the POE only needs the information of the parents and their biological children in each family, we deleted the adopted children in the families and independent individuals from the original data. Finally, we obtained 1,187 families (totally 4,559 individuals), including 621 twin families, 377 full biological sibling families and 189 one-biological-offspring families for this data analysis. See Table 1 for the details.

**TABLE 1:** Samples filtered from original data.

Family type	Original data		Filtered data	
	#Families	#Individuals	#Families	#Individuals
Twin	621	2,484	621	2,484
Full biological sibling	377	1,508	377	1,508
One biological offspring	174	522	189	567
Mixed offspring	15	60	0	0
All adopted offspring	16	64	0	0
Others	980	2,146	0	0
Total	2,183	6,784	1,187	4,559

All 621 twin families, 377 full biological sibling families and 174 one-biological-offspring families were kept. In addition, 15 adopted offspring in 15 mixed families in original data were filtered, and hence 15 mixed families in original data become 15 one-biological-offspring families in filtered data. Others include incomplete families with at least one of the parents missing.

### Genotypes

There are 527,829 SNPs in the MCTFR dataset. Among them, 515,385 are autosomal SNPs. We used the following quality control criteria to filter the SNPs (McGue et al., 2013): 1) genotype call rate < 99%, 2) minor allele frequency < 1%, and 3) individual call rate < 99%. Since all the filtered data are families, the assumption of Hardy-Weinberg equilibrium is not required. After the quality control, 510,278 autosomal SNPs were included in this data analysis.

### Clinical Phenotypes

**FIGURE 1:**
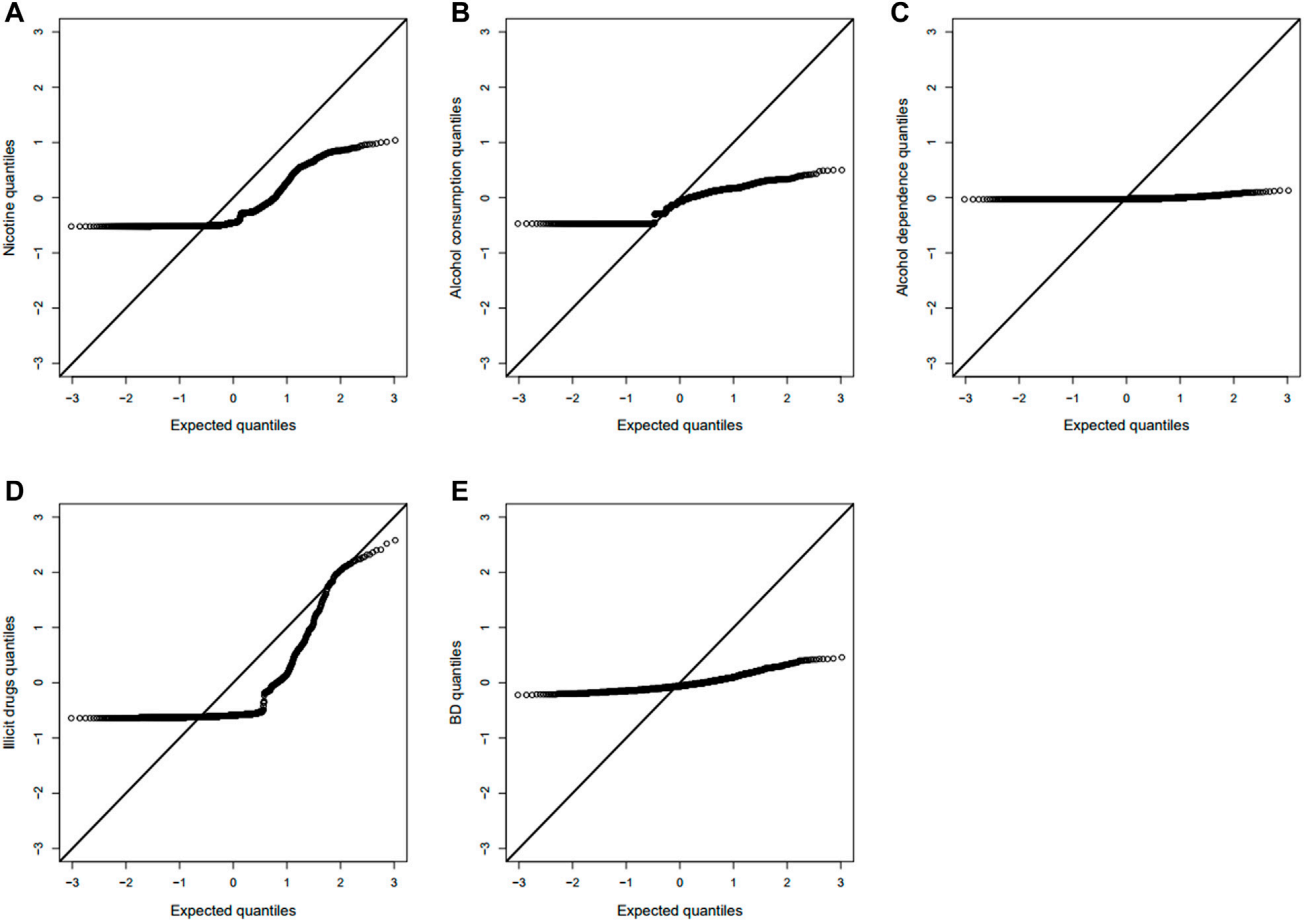
Q-Q plots of five clinical phenotypes (*N* = 1,187). **(A)** Nicotine, **(B)** alcohol consumption, **(C)** alcohol dependence, **(D)** illicit drugs, and **(E)** BD, non-substance use related behavioral disinhibition.

This dataset includes five composite quantitative clinical phenotypes, which are derived using the hierarchical factor analysis method described by Hicks et al. (2011): 1) nicotine (composite score of measures of quantity and frequency of nicotine use and symptoms of nicotine dependence), 2) alcohol consumption (composite score of measures of alcohol use frequency and quantity), 3) alcohol dependence (composite score of diagnostic and statistical manual of mental disorders symptoms of alcohol dependence/abuse and non-diagnostic alcohol-related problems), 4) illicit drugs (composite score of frequency of use of 11 different drug classes and diagnostic and statistical manual of mental disorders symptoms of drug dependence), and 5) BD (composite score of measures of non-substance use related behavioral disinhibition including symptoms of conduct disorder and aggression). Before the analysis, we should firstly carry out the normality tests and the correlation tests for the five phenotypes. We drew the Q-Q plots and performed the Spearman’s rank correlation tests between any two of the five phenotypes based on 1,187 offspring, where only one offspring was randomly selected in each family to remove the correlation between offspring. As shown in Figure 1, we found that none of the five clinical phenotypes follows the normal distribution. There is a significant and high correlation between any two of the phenotypes (ranging from 0.73 to 0.87). See Table 2 for the details. Therefore, to avoid increasing false positive results in the later POE tests at a genome-wide scale, we used the rank-based inverse normal transformation, which was proposed by McCaw et al. (2020), to transform the phenotype data. Then, we conducted the POE tests based on the transformed phenotypes.

**TABLE 2:** Spearman’s rank correlation coefficients among the five clinical phenotypes (*N* = 1,187).

	Nicotine	Alcohol consumption	Alcohol dependence	Illicit drugs	BD
Nicotine	1.00				
Alcohol consumption	0.80	1.00			
Alcohol dependence	0.77	0.83	1.00		
Illicit drugs	0.87	0.87	0.85	1.00	
BD	0.76	0.73	0.75	0.84	1.00

BD, non-substance use related behavioral disinhibition. All the *p*-values of the tests for Spearman’s rank correlation coefficients are less than 0.001.

### Covariates

The models for testing the POE included the covariates age, sex and birth year. Note that the covariate “generation” of the individuals was also collected in the MCTFR dataset. However, the parents in each family were just used to determine the parental origin of minor alleles of their offspring in the POE tests, and then we did not include the covariate “generation” in the models.

### Parent-of-Origin Effects Tests

Each of 510,278 autosomal SNPs was used to test for the POE on each of five transformed clinical phenotypes based on 1,187 families (totally 4,559 individuals). To evaluate the individual contribution of maternal and paternal genetic variations to the phenotypes of the offspring, we used the genotype data from families to determine the parental origin of minor alleles in the offspring. For each given SNP locus, we first needed to construct two indicator variables, the paternally derived minor allele indicator variable (PD) and the maternally derived minor allele indicator variable (MD), which are respectively the number of minor alleles inherited from the father and that from the mother, and take the value of either 0 or 1. When the father, the mother, and the offspring are all heterozygous, we could not identify from whom the minor alleles in the offspring were inherited. So, we referred to the methods proposed by Hochner et al. (2015) and set both PD and MD to be 0.5 (Granot-Hershkovitz et al., 2020). Detailed assignments of the values of the two indicator variables are shown in Table 3. Considering the genetic relationship between the offspring in the families, we used a linear mixed model to test for the POE between each SNP and a phenotype. The model is as follows (Granot-Hershkovitz et al., 2020)

$$Y=\beta_{0}+\beta_{1}\mathrm{PD}+\beta_{2}\mathrm{MD}+\gamma^{T}Z+b+\varepsilon$$ (1)

where Y denotes the phenotype of the offspring, PD is the number of minor alleles inherited from the father in the offspring and MD is the number of minor alleles inherited from the mother in the offspring, and Z is a series of covariates that need to be adjusted, including gender, age and birth year. $\beta_{0}$ is the intercept, $\beta_{1}$ , $\beta_{2}$ and γ are the regression coefficients respectively corresponding to PD , MD and Z . b is a random term which satisfies $b∼N\left(0,C\sigma_{b}^{2}\right)$ , where C is the correlation coefficient matrix between the offspring and the element in the matrix is twice as large as the inbreeding coefficient, and $\sigma_{b}^{2}$ is the variance of the random term; ε is a residual with $\varepsilon ∼N\left(0,I\sigma_{\varepsilon }^{2}\right)$ , where I is the identity matrix, and $\sigma_{\varepsilon }^{2}$ is the variance of the residual. The presence of the POE is assessed *via* the tests with the following null hypotheses: (a) $\beta_{1}=0$ , (b) $\beta_{2}=0$ , and (c) $\beta_{1}=\beta_{2}$ . $\beta_{1}\neq 0$ and $\beta_{1}\neq \beta_{2}$ indicate that there is a paternally derived effect, and $\beta_{2}\neq 0$ and $\beta_{1}\neq \beta_{2}$ mean that there is a maternally derived effect. The *p*-value for the paternally derived effect and that of the maternally derived effect could be obtained directly through the linear mixed model, while the *p*-value for the difference between the paternally derived effect $\beta_{1}$ and the maternally derived effect $\beta_{2}$ was calculated using the F test. The significance levels are as follows: (a) α for testing $\beta_{1}=\beta_{2}$ was set to be 0.05 (Granot-Hershkovitz et al., 2020), and (b) α for testing $\beta_{1}=0$ or $\beta_{2}=0$ was set to be $5\times 10^{-8}$ (Mozaffari et al., 2019).

**TABLE 3:** Indicator variables of parental origin of minor allele.

Father genotype	Mother genotype	Offspring genotype	PD	MD
*AA*	*AA*	*AA*	1	1
*AA*	*Aa*	*AA*	1	1
*AA*	*Aa*	*Aa*	1	0
*AA*	*aa*	*Aa*	1	0
*Aa*	*AA*	*AA*	1	1
*Aa*	*AA*	*Aa*	0	1
*Aa*	*Aa*	*AA*	1	1
*Aa*	*Aa*	*Aa*	0.5	0.5
*Aa*	*Aa*	*aa*	0	0
*Aa*	*aa*	*Aa*	1	0
*Aa*	*aa*	*aa*	0	0
*aa*	*AA*	*Aa*	0	1
*aa*	*Aa*	*Aa*	0	1
*aa*	*Aa*	*aa*	0	0
*aa*	*aa*	*aa*	0	0

Assuming that A is the minor allele.

For comparison, for each candidate SNP, we established another linear mixed model to test for the association between the genotype and the phenotype of the offspring, which does not consider the POE. The model is as follows

$$Y=\beta_{0}+\mathrm{\beta G}+\gamma^{T}Z+b+\varepsilon$$ (2)

where G is the number of minor alleles in the offspring, taking the values of 0, 1 or 2; β is the regression coefficient of G ; other notations have the same meaning as those in model 1. The significance level α for testing β=0 is fixed at $5\times 10^{-8}$ . It should be noted that models 1, 2 only use the data of the offspring.

### Implementation in R

All the above analyses were implemented in the R software (version 4.1.1, http://r-project.org). The linear mixed model was fitted using the “lmekin” function in the R package “coxme.”

## Results

To determine the parental origin of the minor alleles in offspring, we only selected those complete families for analysis. Therefore, we used 1,187 families (4,559 individuals) in the filtered data of Table 1, of which 2,374 were parents and 2,185 were offspring. The mean and the standard deviation (SD) of the age of the fathers were 44.6 ± 5.4 years and those of the mothers were 42.4 ± 4.9 years. There were 992 (45.4%) males in the offspring generation with the mean and the SD of the age being 17.8 ± 0.6 years, and 1,193 (54.6%) females with the mean and the SD of the age being 17.9 ± 0.8 years.

**FIGURE 2:**
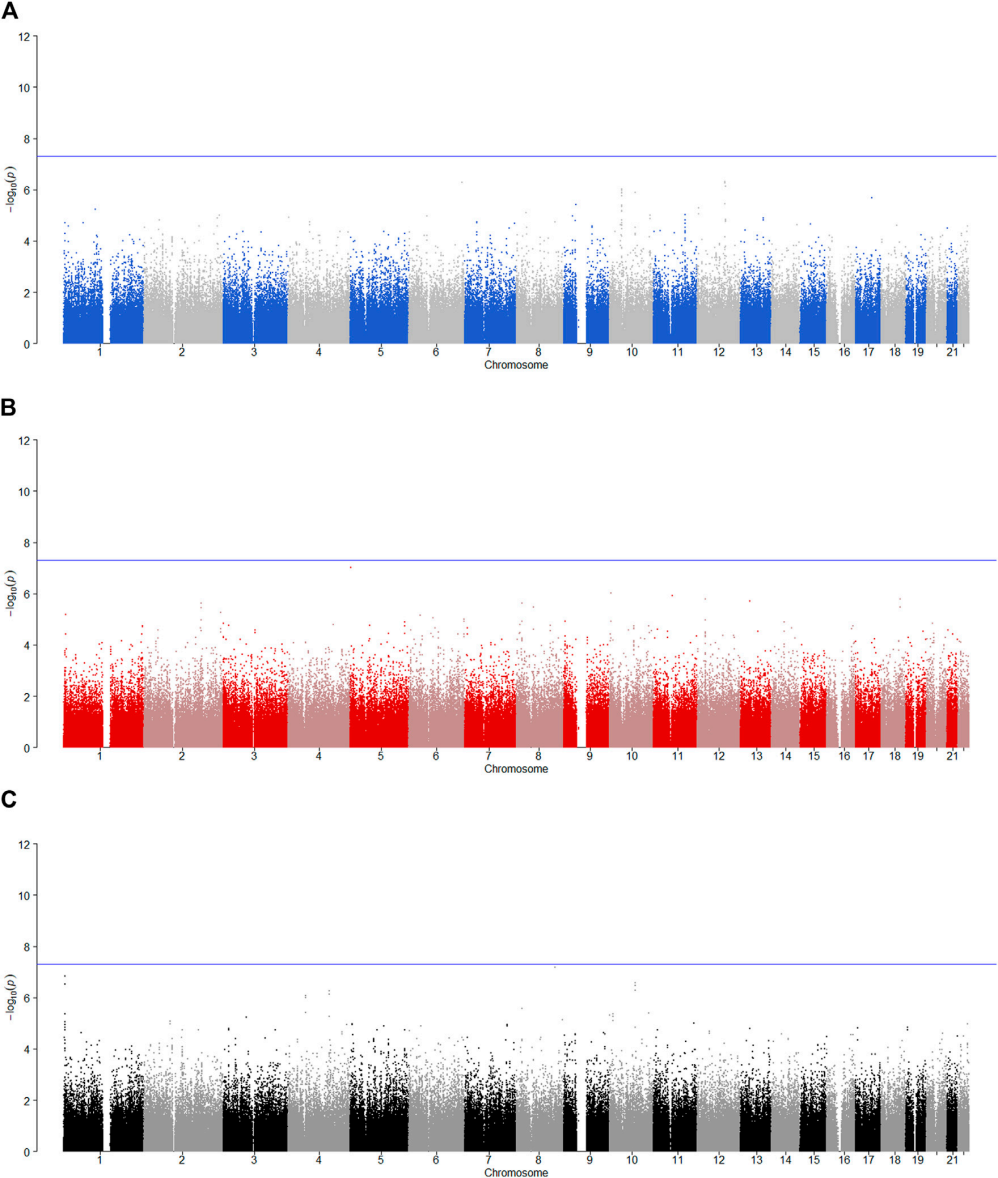
Manhattan plot for POE and genotypic effect tests of nicotine (*N* = 2,185). The blue line represents the significance level $\alpha =5\times 10^{-8}$ , which is the significance level of GWAS. **(A)** Paternally derived effect, **(B)** maternally derived effect and **(C)** genotypic effect.

**FIGURE 3:**
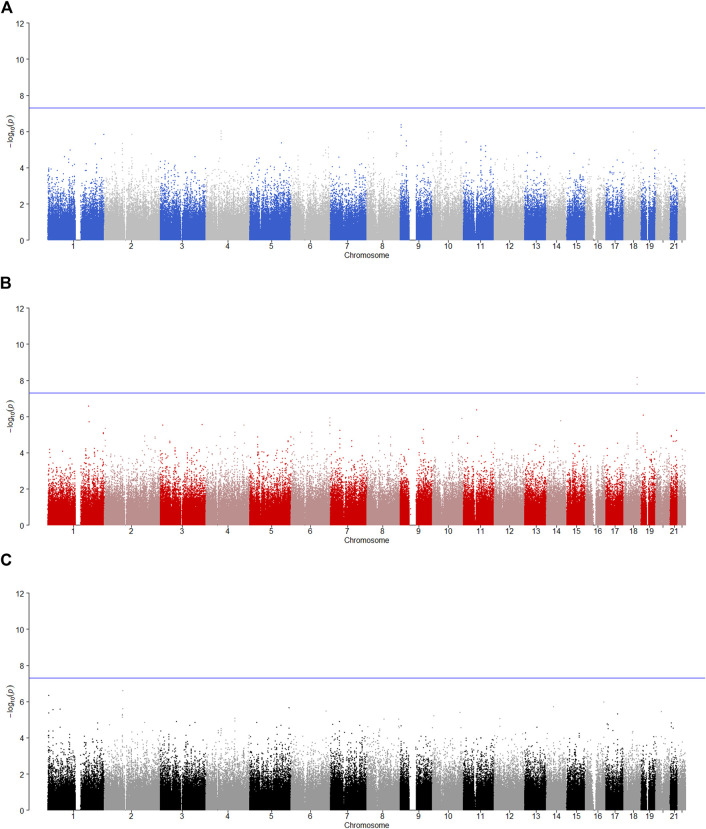
Manhattan plot for POE and genotypic effect tests of alcohol consumption (*N* = 2,185). The blue line represents the significance level $\alpha =5\times 10^{-8}$ , which is the significance level of GWAS. **(A)** Paternally derived effect, **(B)** maternally derived effect and **(C)** genotypic effect.

**FIGURE 4:**
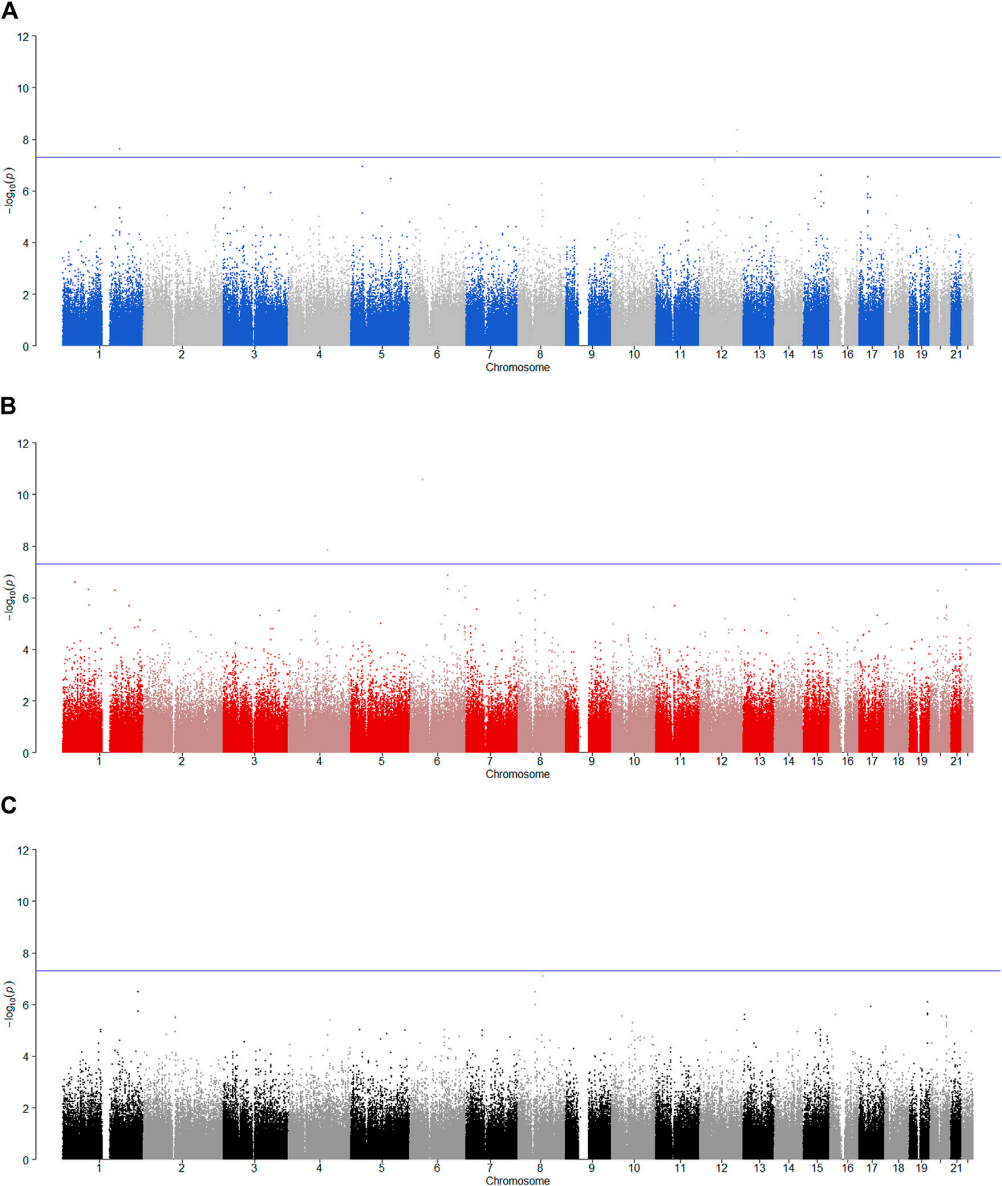
Manhattan plot for POE and genotypic effect tests of alcohol dependence (*N* = 2,185). The blue line represents the significance level $\alpha =5\times 10^{-8}$ , which is the significance level of GWAS. Note that three SNPs on Chromosome 6 are overlapped. **(A)** Paternally derived effect, **(B)** maternally derived effect and **(C)** genotypic effect.

**FIGURE 5:**
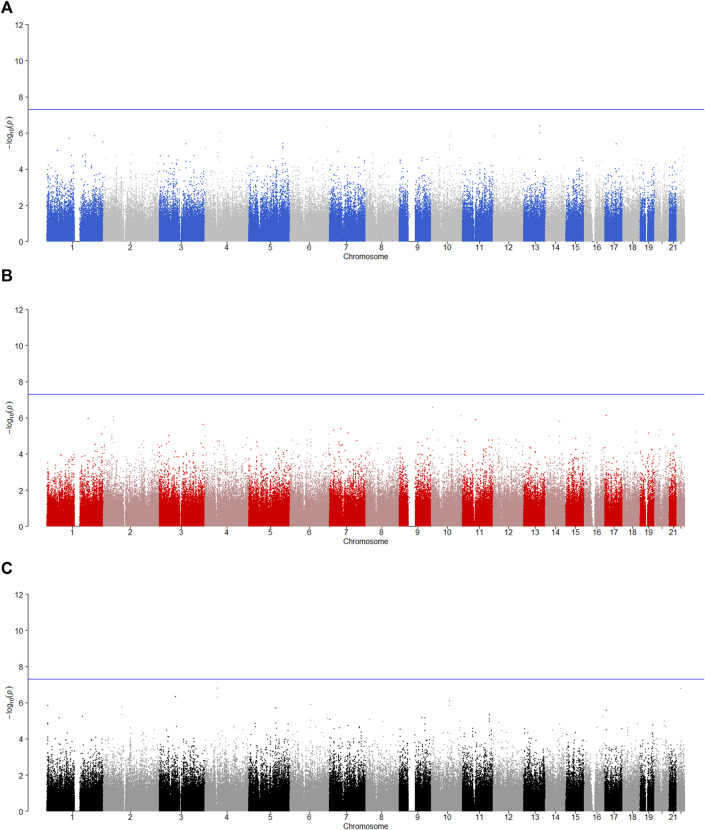
Manhattan plot for POE and genotypic effect tests of illicit drugs (*N* = 2,185). The blue line represents the significance level $\alpha =5\times 10^{-8}$ , which is the significance level of GWAS. **(A)** Paternally derived effect, **(B)** maternally derived effect and **(C)** genotypic effect.

**FIGURE 6:**
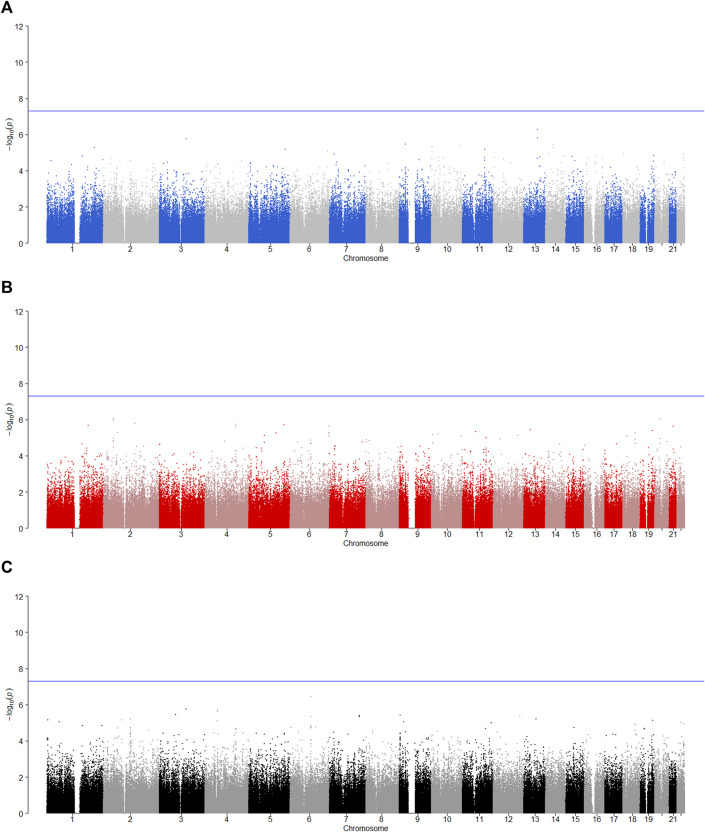
Manhattan plot for POE and genotypic effect tests of non-substance use related behavioral disinhibition (*N* = 2,185). The blue line represents the significance level $\alpha =5\times 10^{-8}$ , which is the significance level of GWAS. **(A)** Paternally derived effect, **(B)** maternally derived effect and **(C)** genotypic effect.

We tested the POE between 510,278 autosomal SNPs and five phenotypes by model 1 for 2,185 offspring. The significance levels for testing $\beta_{1}=\beta_{2}$ and for testing $\beta_{1}=0$ or $\beta_{2}=0$ were respectively set to be 0.05 and $5\times 10^{-8}$ . As such, when the *p*-value of testing for $\beta_{1}=0$ is less than $5\times 10^{-8}$ and the *p*-value of testing for $\beta_{1}=\beta_{2}$ is less than 0.05, the SNP has the paternally derived effect (i.e., maternal imprinting). When the *p*-value of testing for $\beta_{2}=0$ is less than $5\times 10^{-8}$ and the *p*-value of testing for $\beta_{1}=\beta_{2}$ is less than 0.05, the SNP has the maternally derived effect (i.e., paternal imprinting). Figures 2–6 give the Manhattan plots of the POE tests and genotypic effect test for the five phenotypes. We finally identified 9 SNPs which show the statistically significant POE, of which rs704833, rs6837925, rs4141854, rs9394515, rs4711553, rs1863548, and rs11067062 showed significant POE on alcohol dependence; rs12960235 and rs715351 showed significant POE on alcohol consumption. Table 4 gives the corresponding results, which also includes the estimates of the effect sizes.

**TABLE 4:** Parent-of-origin effects tests for five phenotypes.

SNP	CHR	Position	Gene	Minor allele	MAF	Pheno-type	Paternally derived effect			Maternally derived effect			Testing $\beta_{1}=\beta_{2}$		
							$\beta_{1}$	CI	*p*-value	$\beta_{2}$	CI	*p*-value	$\beta_{1}-\beta_{2}$	CI	*p*-value
rs704833	1	176216923		*G*>*A*,*C*,*T*	0.081	DEP	0.012	(0.001, 0.023)	**2.442E-08**	0.001	(−0.010, 0.012)	6.816E-01	0.011	(0.005, 0.017)	**2.454E-04**
rs6837925	4	119073195		*T*>*C*	0.002	DEP	4.228E-04	(−0.055, 0.055)	9.654E-01	0.080	(0.004, 0.156)	**1.462E-08**	−0.080	(−0.113, −0.047)	**3.236E-06**
rs4141854	6	38539736	*BTBD9*	*T*>*C*	0.002	DEP	−0.003	(−0.047, 0.041)	7.160E-01	0.133	(0.024, 0.242)	**2.797E-11**	−0.136	(−0.177, −0.095)	**2.060E-10**
rs9394515	6	38552483	*BTBD9*	*G*>*A,T*	0.002	DEP	−0.003	(−0.047, 0.041)	7.160E-01	0.133	(0.024, 0.242)	**2.797E-11**	−0.136	(−0.177, −0.095)	**2.060E-10**
rs4711553	6	38641724	*BTBD9*	*C*>*A,T*	0.001	DEP	−0.003	(−0.052, 0.046)	7.481E-01	0.133	(0.024, 0.242)	**2.790E-11**	−0.136	(−0.179, −0.093)	**5.444E-10**
rs1863548	12	114317201		*C*>*T*	0.004	DEP	0.045	(0.001,0.089)	**4.511E-09**	−0.002	(−0.040, 0.036)	8.061E-01	0.047	(0.025, 0.069)	**7.993E-06**
rs11067062	12	114344341		*G*>*A*	0.003	DEP	0.051	(0.002, 0.100)	**3.117E-08**	0.005	(−0.050, 0.060)	6.528E-01	0.046	(0.019, 0.073)	**8.066E-04**
rs12960235	18	63586256	*SERPINB13*	*A*>*G*	0.253	CON	−0.079	(−0.341, 0.188)	9.750E-02	0.275	(0.008, 0.542)	**1.632E-08**	−0.354	(−0.493, −0.215)	**7.107E-07**
rs715351	18	63588207	*SERPINB13*	*T*>*C*	0.267	CON	−0.053	(−0.304, 0.198)	2.487E-01	0.281	(0.019, 0.543)	**7.090E-09**	−0.334	(−0.471, −0.197)	**2.087E-06**

CHR, chromosome of SNP; MAF, minor allele frequency; DEP, alcohol dependence; CON, alcohol consumption; CI, confidence interval. Entries in bold indicate statistically significant results.

SNP rs704833 is located on Chromosome 1 and the results demonstrate that there is a significant POE at rs704833 on alcohol dependence. The minor allele inherited from the father has the significant association with alcohol dependence ( $\beta_{1}=0.012$ , *p*-value = $2.442\times 10^{-8}$ ) and the *p*-value for testing $\beta_{1}=\beta_{2}$ is $2.454\times 10^{-4}$ . However, the corresponding maternally derived effect is not statistically significant ( $\beta_{2}=0.001$ , *p*-value = $6.816\times 10^{-1}$ ). This means that SNP rs704833 has maternal imprinting. Meanwhile, due to $\beta_{1}>0$ , the minor allele at rs704833 is a risk allele, which will increase the degree of alcohol dependence. SNP rs6837925, located on Chromosome 4, has a significant maternally derived effect on alcohol dependence ( $\beta_{2}=0.080$ , *p*-value = $1.462\times 10^{-8}$ ) and the *p*-value for testing $\beta_{1}=\beta_{2}$ is $3.236\times 10^{-6}$ , while its paternally derived effect is not significant ( $\beta_{1}=4.228\times 10^{-4}$ , *p*-value = $9.654\times 10^{-1}$ ), indicating that there is paternal imprinting at rs6837925. Three SNPs on Chromosome 6, rs4141854, rs9394515, and rs4711553 are all located in the *BTBD9* gene, and have significant maternally derived effect on alcohol dependence, where the corresponding *p*-values at these three SNPs are respectively $2.797\times 10^{-11}$ , $2.797\times 10^{-11}$ and $2.790\times 10^{-11}$ . None of them has significant paternally derived effect (all the *p*-values being larger than $5\times 10^{-8}$ ), which indicates that there is paternal imprinting at these three loci. Furthermore, for these three SNPs, the regression coefficients $\beta_{2}$ ’s are all 0.133 which are greater than 0 and the *p*-values for testing $\beta_{1}=\beta_{2}$ are respectively $2.060\times 10^{-10}$ , $2.060\times 10^{-10}$ and $5.444\times 10^{-10}$ . Therefore, the minor allele at these loci would increase people’s dependence on alcohol. It can be seen that the results at SNPs rs4141854 and rs9394515 are completely consistent. Comparing their positions on the chromosome, these two SNPs are close to each other, so they may be linked. Two SNPs on Chromosome 12, rs1863548 and rs11067062, have significant paternally derived effect on alcohol dependence (all the *p*-values $<5\times 10^{-8}$ ) and all the *p*-values for testing $\beta_{1}=\beta_{2}$ are less than 0.05. From Table 4, the maternally derived effects of these two SNPs are not significant (all the *p*-value > $5\times 10^{-8}$ ). At the same time, both the $\beta_{1}$ ’s are bigger than 0, so the minor alleles at these two loci are a harmful factor for alcohol dependence, i.e., the minor alleles will increase the risk of alcohol dependence. Finally, SNPs rs12960235 and rs715351 are both included in the *SERPINB13* gene on Chromosome 18, the regression coefficients of MD ( PD ) on alcohol consumption are $\beta_{2}=0.275$ ( $\beta_{1}=-0.079$ ) and $\beta_{2}=0.281$ ( $\beta_{1}=-0.053$ ), respectively, with the respective *p*-values being $1.632\times 10^{-8}$ ( $9.750\times 10^{-2}$ ) and $7.090\times 10^{-9}$ ( $2.487\times 10^{-1}$ ). The *p*-values for testing the maternally derived effect are all less than $5\times 10^{-8}$ , and the *p*-values for testing the paternally derived effect are all greater than $5\times 10^{-8}$ , which means that SNPs rs12960235 and rs715351 both have significant maternally derived effect (i.e., paternal imprinting). Note that $\beta_{2}$ ’s are larger than 0, so the minor alleles at these two loci will increase alcohol consumption.

For comparison, Table 5 displays the results of genotypic effect tests for the above-mentioned 9 SNPs with the POE based on model 2. It is shown in Table 5 that model 2 could not identify the association between the SNPs and the phenotypes at the significance level of $5\times 10^{-8}$ , when directly using the genotypes and not considering the POE information in association analysis. However, after incorporating the POE based on model 1, all the nine SNPs can be found. For example, when using model 2 to explore the association between SNP rs704833 and alcohol dependence, the *p*-value of $2.520\times 10^{-5}$ is not statistically significant, while model 1 suggests the significant paternally derived effect (*p*-value = $2.442\times 10^{-8}$ ) if we consider the POE. Meanwhile, by comparing the corresponding regression coefficients of two models for SNP rs704833, we could see that the signs of β and $\beta_{1}$ are the same, but the absolute value of β is smaller than the absolute value of $\beta_{1}$ . Therefore, when exploring the influence of SNPs on phenotypes, the incorporation of the POE can improve the test power of the model.

**TABLE 5:** Genotypic effect test for five phenotypes.

SNP	CHR	Position	Gene	Minor allele	MAF	Phenotype	Genotypic effect		
							β	CI	*p*-value
rs704833	1	176216923		*G*>*A*,*C*,*T*	0.081	DEP	0.006	(−0.005, 0.017)	2.520E-05
rs6837925	4	119073195		*T*>*C*	0.002	DEP	0.026	(−0.018, 0.070)	1.306E-03
rs4141854	6	38539736	*BTBD9*	*T*>*C*	0.002	DEP	0.014	(−0.024, 0.052)	4.631E-02
rs9394515	6	38552483	*BTBD9*	*G*>*A,T*	0.002	DEP	0.014	(−0.024, 0.052)	4.631E-02
rs4711553	6	38641724	*BTBD9*	*C*>*A,T*	0.001	DEP	0.020	(−0.024, 0.064)	1.593E-02
rs1863548	12	114317201		*C*>*T*	0.004	DEP	0.020	(−0.007, 0.047)	1.479E-04
rs11067062	12	114344341		*G*>*A*	0.003	DEP	0.031	(−0.007, 0.069)	1.008E-05
rs12960235	18	63586256	*SERPINB13*	*A*>*G*	0.253	CON	0.094	(−0.080, 0.268)	3.691E-03
rs715351	18	63588207	*SERPINB13*	*T*>*C*	0.267	CON	0.107	(−0.067, 0.281)	8.197E-04

CHR, chromosome of SNP; MAF, minor allele frequency; DEP, alcohol dependence; CON, alcohol consumption; CI, confidence interval.

## Discussion

Behavioral disinhibition could easily cause great harm to human health and social stability. At present, the research on the influencing factors of behavioral disinhibition has gone further to the genetic level. However, the existing studies have not considered the POE in the analysis, which would miss or underestimate the genetic influence of certain loci on behavioral disinhibition. Therefore, in this study, we explored the POE of genetic loci on behavioral disinhibition based on the MCTFR data. Specifically, we first filtered the MCTFR data and obtained nuclear family data we needed, and then performed the normality tests of the five phenotypes closely related to behavioral disinhibition in the MCTFR data. Note that all the five phenotypes do not follow normal distributions. As such, we used the rank-based inverse normal transformation on the phenotypes to avoid increasing false positive results. Then, the method proposed in Hochner et al. (2015) was utilized to carry out the POE tests for all 510,278 autosomal SNPs at a genome-wide level, and finally nine SNPs were identified to have the significant POE on behavioral disinhibition.

Among the nine identified SNPs, seven SNPs have statistically significant POE on alcohol dependence and two SNPs have statistically significant POE on alcohol consumption. SNPs rs4141854, rs9394515, and rs4711553 on Chromosome 6, which have statistically significant POE on alcohol dependence, were located in the same gene, *BTBD9*. The *BTBD9* gene is located on the short arm of Chromosome 6 and is highly expressed throughout the human brain (Freeman et al., 2012), and its mutation has been shown to be associated with restless legs syndrome and Tourette’s syndrome (Rivière et al., 2009; Schormair et al., 2017; Lyu et al., 2019). Restless legs syndrome is a pervasive chronic neurological disorder that causes nerve impulses to stimulate leg muscle twitching uncontrollably at night and at rest (Schormair et al., 2017). Tourette’s syndrome is similar to restless legs syndrome, except that nerve impulses are transferred from the legs to the face (Rivière et al., 2009). Studies have shown that due to the uncontrollable nerve impulses of these two diseases at night, two prominent symptoms of the diseases are the insomnia and the severely reduced quality of life, and the latter leads to a significant increase in the risk of depression, anxiety and alcoholism (Allen et al., 2011; Blaty and DelRosso, 2022). Studies have also shown that insomnia and alcohol dependence are significantly positively correlated (Chakravorty et al., 2016). Regarding the POE of Tourette’s syndrome, some studies have pointed out that the age of the onset of the maternally derived offspring is earlier than that of the paternally derived offspring significantly, which suggests that the maternally derived effect of the *BTBD9* gene on Tourette’s syndrome could be explained by meiotic events or even intrauterine environmental influences (Eapen et al., 1997), and this supports our findings. The remaining four SNPs rs704833, rs6837925, rs1863548, and rs11067062 having statistically significant POE on alcohol dependence do not belong to any gene, and there has been no study to explain the functions of these four SNPs. These novel SNPs we have discovered may be associated with behavioral disinhibition, but this still needs to be confirmed by subsequent molecular genetics.

SNPs rs12960235 and rs715351 on Chromosome 18 have statistically significant POE on alcohol consumption and belong to the *SERPINB13* gene. The *SERPINB13* gene is a protein-coding gene, and the protein function annotations associated with this gene include serine-type endopeptidase inhibitor activity and cysteine-type endopeptidase inhibitor activity. The *SERPINB13*-related diseases include head and neck squamous cell carcinoma (de Koning et al., 2009), skin cancer (Moussali et al., 2005) and type I diabetes (Kryvalap et al., 2021). However, this gene has not been found to be associated with alcohol consumption in literature, and only studies have shown that alcoholism is a risk factor for diabetes (Tetzschner et al., 2018). However, we think that this is a normal phenomenon, because few people have considered the POE in the previous association studies, which may reduce the test power of the GWAS and make some positive SNPs not detected. On the other hand, McGue et al. (2013) conducted a GWAS on the basis of the MCTFR data and found a significant SNP rs1868152 (*p*-value = $4.9\times 10^{-8}$ ) related to illicit drugs. However, this SNP has not been identified in our study. The possible reasons may be as follows. First, the sample used in our study is different from that of McGue et al. (2013). Specifically, we only used biological offspring in families, while McGue et al. (2013) used both biological offspring and adopted offspring in families and independent individuals in the MCTFR data. Second, we performed a rank-based inverse normal transformation on the five phenotypes to avoid increasing false positive results, while McGue et al. (2013) used the original five phenotypes as the outcome variables. Third, McGue et al. (2013) did not consider the POE in the GWAS.

We searched the imprinted-gene database (http://igc.otago.ac.nz/) constructed by Morison et al. (2001) and the geneimprint and Otago imprint databases (https://www.geneimprint.com/), while the 9 SNPs with the POE identified in our study are not included in these databases. Strauch et al. (2005) conducted a parametric single-marker linkage analysis and the POE test on alcohol dependence and alcohol consumption data of 93 Caucasian pedigrees composed of 919 people. The results showed that paternally derived effects were found on Chromosome 7, and maternally derived effects were found on Chromosomes 1, 2, 10, 13, 15 and 21. Another study based on alcoholism data (Liu et al., 2005) found the evidence of paternally derived effects on Chromosome 7 and maternally derived effects on Chromosomes 10 and 12. In our study, we found the paternally derived effects on Chromosomes 1 and 12, and the maternally derived effects on Chromosomes 4, 6, and 18. This may suggest that there is the POE on behavioral disinhibition, especially alcohol dependence and alcohol consumption.

We used two models to conduct the association analysis in Tables 4, 5, respectively with the POE and without the POE, to compare the performances of these two models at the nine SNPs. It can be seen from Table 5 that model 2 not considering the POE does not find any significant SNPs among the nine SNPs. This suggests that if the POE is present but is not considered in the association analysis, the genetic factors of the phenotypes may be ignored or underestimated. Granot-Hershkovitz et al. (2020) and Nudel et al. (2014) have also drawn similar conclusions in their studies. On the other hand, there are several limitations in our study. First, the MCTFR dataset only contains a few covariates (age, sex, birth year and generation). Although we adjusted them (except for the covariate generation) in the model, we are not sure if we have considered most of the variances of environmental factors. Second, the POE is closely related to methylation while we did not include any methylation data in the analysis. To obtain more significant results, methylation data should be incorporated into the detection of the POE in future. In addition, there has been a novel method available for inferring the POE in literature (Hofmeister et al., 2021), which does not require parental genomes nor prior knowledge of genealogy. As such, it is suitable for many data types, besides nuclear families with both parents. We will use this method to reanalyze the MCTFR data for the POE on behavioral disinhibition in future. Finally, this study has the following contributions. 1) Facing the challenge that the five behavioral inhibition phenotypes do not follow normal distributions, we used the rank-based inverse normal transformation method on the five phenotypes to avoid increasing false positive results. 2) Verify the conclusion proposed by Granot-Hershkovitz et al. (2020) that taking account of the POE in GWAS can enhance the ability to detect genetic association and population characteristics. 3) Provide novel evidence for the presence of the POE on behavioral disinhibition.

## Data Availability

The Minnesota Center for Twin and Family Research data used for this study can be found on the database of Genotypes and Phenotypes with accession number 86747-6 (https://www.ncbi.nlm.nih.gov/projects/gap/cgi-bin/study.cgi?study_id=phs000620.v1.p1.
